# Improved Frequency Domain Turbo Equalization with Expectation Propagation Interference Cancellation in Underwater Acoustic Communications

**DOI:** 10.3390/s23187801

**Published:** 2023-09-11

**Authors:** Bin Jiang, Yue Tang, Yinan Zhao, Jianrong Bao, Chao Liu, Xianghong Tang

**Affiliations:** 1 School of Communication Engineering, Hangzhou Dianzi University, Hangzhou 310018, China; jiangbin@hdu.edu.cn (B.J.); tx@hdu.edu.cn (Y.T.); baojr@hdu.edu.cn (J.B.); liuchao@hdu.edu.cn (C.L.); tangxh@hdu.edu.cn (X.T.); 2 School of Electronics & Information, Hangzhou Dianzi University, Hangzhou 310018, China; 3 Sichuan Provincial Energy Investment Group Co., Ltd., Chengdu 610041, China; 4 National Mobile Communications Research Laboratory, Southeast University, Nanjing 210096, China

**Keywords:** underwater acoustic communication, expectation propagation, adaptive sparse channel estimation, a posteriori soft decision, frequency domain turbo equalization

## Abstract

This paper proposes an improved frequency domain turbo equalization (IFDTE) with iterative channel estimation and feedback to achieve both a good performance and low complexity in underwater acoustic communications (UWACs). A selective zero-attracting (SZA) improved proportionate normal least mean square (SZA-IPNLMS) algorithm is adopted by utilizing the sparsity of the UWAC channel to estimate it using a training sequence. Simultaneously, a set-membership (SM) SZA differential IPNLMS (SM SZA-DIPNLMS) with variable step size is adopted to estimate the channel status information (CSI) in the iterative channel estimation with soft feedback. In this way, the computational complexity for iterative channel estimation is reduced effectively with minimal performance loss. Different from traditional schemes in UWACs, an IFDTE with expectation propagation (EP) interference cancellation is adopted to estimate the a posteriori probability of transmitted symbols iteratively. A bidirectional IFDTE with the EP interference cancellation is proposed to further accelerate the convergence. THe simulation results show that the proposed channel estimation obtains 1.9 and 0.5 dB performance gains, when compared with those of the IPNLMS and the $l_{0}$-IPNLMS at a bit error rate (BER) of $10^{-3}$. The proposed channel estimation also effectively reduces the unnecessary updating of the coefficients of the UWAC channel. Compared with traditional time-domain turbo equalization and FDTE in UWACs, the IFDTE obtains 0.5 and 1 dB gains in the environment of SPACE’08 and it obtains 0.5 and 0.4 dB gains in the environment of MACE’04 at a BER of $10^{-3}$. Therefore, the proposed scheme obtains a good BER performance and low complexity and it is suitable for efficient use in UWACs.

## 1. Introduction

Underwater acoustic communications (UWACs) have been widely used as sensing and communication techniques in underwater sound measurement and fishing. They are adequately developed and equipped in the underwater sensors of ships, submarines, autonomous underwater vehicles, and so on. However, UWAC channels are time-varying and multipath-fading. The received signals usually have serious Doppler distortion and inter-symbol interference [1]. Their high-quality channel recovery has attracted wide attention in the literature. Turbo equalization is used to effectively recover original transmission signals by exchanging the extrinsic information between an equalizer and a decoder iteratively [2]. Many schemes of turbo equalization were proposed for UWACs in the past years. The first Turbo equalization applied to UWACs was a joint maximum a posteriori probability (MAP) equalization [3]. Although the pre-survivor processing was only adopted to track the most likely path of trellis states for MAP equalization, the computational complexity remains high. Consequently, the design of low complexity turbo equalization based on a minimum mean square error (MMSE) was proposed. Time-domain turbo equalization (TDTE) in UWACs extending conventional MMSE linear equalization or soft decision feedback equalization (SDFE) was reported in the literature [4,5]. The receivers exhibit good performance with a computational complexity quadratic scaling at the block length and the length of finite channel impulse response (CIR). The length of the filter for equalization is longer than the UWAC channels, and the lengths of UWAC channels are often longer than 50 [2]. Therefore, the time-domain turbo equalization still experiences huge computational complexity.

A linear frequency domain turbo equalization (FDTE) was used in UWACs to initially process the received signals and to reduce the computational complexity of time-domain turbo equalization [6]. In the FDTE, the parallel block equalization filters were implemented using fast Fourier transformation (FFT) with much efficient computation of parallel convolution; it thus improved the computational complexity significantly by reducing many multiplications in the filtering calculations of the TDTE. In addition, some more techniques, such as the selective zero attracting penalty, selective update strategy, bidirectional structure, and so on, can be combined closely in the FDTE to further improve the performance. Good performance was obtained in low-order modulation. However, the performance degraded radically due to severe Doppler shift under high-order modulation. The performance gap between the FDTE and TDTE still exists. Consequently, nonlinear FDTE was applied in UWACs to improve the performance of receivers. An improved FDTE (IFDTE) with interference cancellation and phase-locked loop was applied in UWACs [7]. Although it effectively overcame the phase ambiguity problem and achieved better performance compared with linear FDTE, its limitation was the high complexity of multiple layers for the equalization of symbols. A frequency domain decision feedback FDTE (FDDF-FDTE) scheme with iterative channel estimation was adopted to achieve a good trade-off between performance and complexity; its feasibility was then verified in terms of the SPACE’08 sea trial data [8]. Coarse estimation based on the Bayesian principle was utilized to estimate the a posteriori probability of transmitted symbols and to enhance the performance of the FDDF-FDTE [9]. Therefore, the turbo equalization with the estimation of a posteriori distribution is important in practice.

Expectation propagation (EP) was widely used as the machine learning for signal detection, using simple distributions to approximate complex ones. The block-EP [10], EP-Filter [11], and the DFE-IC EP [12] had outperformed traditional algorithms under the MMSE criterion. The complexity is relatively higher than that of the traditional TDTE due to the self-iteration of the EP. The FDTE based on the EP was applied for multiuser detection with the known channel to reduce the complexity [13]. To the best of our knowledge, no detailed investigation was conducted on turbo equalization based on the EP in UWACs. Iterative channel estimation can effectively shorten the necessary length of the training sequence for convergence because of the band-limited nature of the UWAC channel, this process improves the efficiency of the spectrum utilization [14,15,16,17]. However, computational complexity inevitably increases due to the reuse of soft information. Adaptive channel estimation was widely used in UWAC channel estimation due to its simple structure and minimal calculation. However, its accuracy was slightly worse than those of compressed sensing (CS) [18].

The CS-based channel estimation updated tap coefficients through heuristic search and inverse operations, resulting in high computational complexity. The combination of norm constraint and adaptive algorithm can achieve rapid convergence with minimal computation [19]. A SZA normal least mean square (NLMS) (SZA-NLMS) algorithm, belonging to the $l_{1}$-norm constrained adaptive algorithm, directly set the tap coefficients below the threshold to zero. Under other similar $l_{1}$-norm constrained channel estimations, it had faster convergence and better signal recovery performance than that of the standard NLMS equalization [20,21]. Recently, to effectively combat the selective fading of underwater acoustic channels for single-carrier deep-sea vertical acoustic communications, an improved proportionate normalized minimum-SER (IPNMSER) algorithm was proposed for adaptive turbo equalization by utilizing the minimum-SER (MSER) criterion to minimize the system’s SER directly [22]. Also, a hybrid frequency–time domain turbo equalizer (FTD-TEQ) was proposed to benefit from two turbo equalizers to solve the slow-convergence problem at different iterative stages for UWACs with comprehensive experimental investigations [23]. Furthermore, the block implementation of least mean square based frequency domain direct adaptive turbo equalization was proposed for use in UWACs [24] and it can be combined with excellent Shannon capacity, approaching channel codes for the better performance and security of turbo equalization. Then, security-oriented Polar coding can be adopted based on channel-gain-mapped frozen bits [25]. And decoding algorithms for low-density parity-check (LDPC) codes were proposed [26] and they can be used for such applications in turbo equalization.

According to the aforementioned analyses in the literature, efficient turbo equalization with both good performance and low complexity is the exact challenge in UWACs due to the fast time variance fading in UWAC channels. The limitations of existing equalization schemes means they usually do not make full use of signals, especially the more accurate equalization metrics of the a posteriori probability of transmitted symbols. In addition, the EP interference cancellation is not employed for better equalization performance. Then, the above two aspects motivate the need for an improved approach for further improvement in turbo equalization. In this paper, an improved FDTE (IFDTE) with the EP interference cancellation for iterative channel estimation is proposed to promote the performance of signal recovery with low complexity in UWACs. This scheme is studied in a single-input multiple-output (SIMO) system. Its performance improvement and complexity reduction are analyzed and verified through numerical simulations. Finally, the main contributions are summarized as follows.

- A precise sparse adaptive channel estimating using the selective zero attracting penalty term.

A SZA-IPNLMS is extended to estimate the channel state information (CSI) in terms of training sequences. Compared with the past sparse adaptive channel estimation, the SZA-IPNLMS gives different constraints to update channel coefficients in accordance with the ratio of channel coefficients to the maximum one. Thus, small channel coefficients are preserved well, and precise CSI is obtained.

- Computational complexity reduction with minimal performance loss using the selective update strategy.

Compared with traditional iterative channel estimation in UWACs, a threshold is set to selectively update the coefficients with a large offset in terms of the estimated noise variance using the SZA-IPNLMS. Thus, it effectively reduces the unnecessary updating of the channel coefficients. A SZA-DIPNLMS is adopted to replace the SZA-IPNLMS for reducing the counts of update operations for the proportionate step matrix through a fixed update period. The step size is dynamically set in accordance with the noise variance and offset of the estimation to maintain a good performance. In this way, the computational complexity of the iterative channel estimation is effectively reduced with a small bit error rate (BER) loss.

- High-quality recovery of UWAC signals by utilizing the IFDTE and bidirectional structure of the equalization.

The IFDTE is applied to UWACs combined with iterative channel estimation. Different from the traditional equalization used in UWACs, the IFDTE obtains a precise a posteriori probability of the transmitted symbols by estimating them iteratively based on the EP. In this way, the IFDTE achieves good performance of the UWAC signal recovery with trade-off complexity compared with those of traditional FDTEs. A bidirectional structure of equalization is utilized to acquire the bidirectional gain to promote the performance of the IFDTE. Thus, the bidirectional IFDTE (Bi-IFDTE) achieves a better performance than the IFDTE. Because of the reliable a posteriori estimation of symbols obtained by the EP, symbols mapped based on an a posteriori estimation served as the training sequence to improve the performance of the channel estimation. Through the high precise channel and symbols, the estimation improves the performance of the interference cancellation in the equalization to achieve a high-quality recovery of UWAC signals.

The organization of this paper is briefly introduced as follows. Section 2 presents a SIMO model of UWACs. Section 3 introduces sparse adaptive channel estimation with a SZA term. A low complexity iterative channel estimation with a selective update of channel coefficients is proposed. Different from the traditional turbo equalization in UWACs, the IFDTE based on the EP is introduced in detail to estimate the actual a posteriori distribution precisely. A bidirectional structure of equalization is proposed to further enhance the performance of the IFDTE. The complexity of the IFDTE is analyzed in this part. The computational complexity of the IFDTE is slightly higher than the traditional FDTE but lower than the traditional TDTE. Section 4 analyses the MSE, BER performance, and computational complexity of the proposed channel estimation scheme. Extrinsic information transfer (EXIT) charts and BER curves are applied jointly for numerical simulations to verify the good performance of convergence and signal recovery. The results show that the proposed scheme outperforms other existing equalization schemes. Section 5 concludes the whole paper. The list of abbreviations used in this paper is shown in the abbreviations for clarity.

## 2. SIMO System Model in UWACs

**Figure 1:**
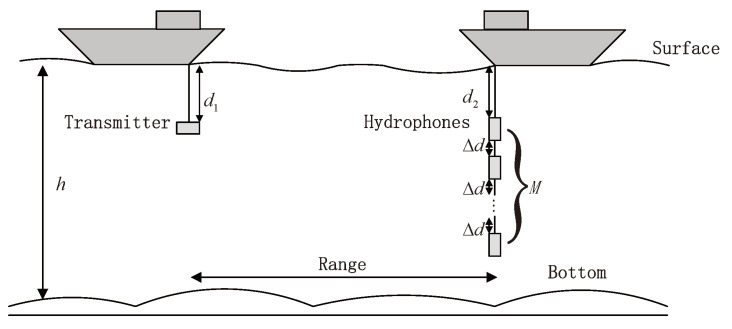
UWACs system model.

Suppose a binary bit sequence $b=[b_{0},b_{1},\ldots ,b_{N_{b}-1}]$ is emitted in the transmitter, where the $N_{b}$ is the length of the bit sequence. This sequence is denoted as “0” or “1” by channel encoding with binary code bit c. The coded bits are interleaved and then mapped to the sequence of symbols $x=\left[x_{1},x_{2},\ldots ,x_{K}\right],x_{k}\in A$, where $x_{k}\in A$, A is a set of mapping symbols, and $x_{k}$ is mapped by Q bit coded bits $c_{k}={\left[c_{k,1},c_{k,2},\ldots ,c_{k,Q}\right]}^{T}$, where $c_{k,q}\in \left\{0,1\right\}$. Subsequently, the training sequence is then inserted into the front of the information blocks. The blocks are modulated to carriers, transmitted through UWAC channels, and received by a hydrophone array, as shown in Figure 1.

The UWAC channels between the transmitter and hydrophones are time-varying. Each symbol block remains unchanged, and the channel varies randomly between different blocks. After synchronization and sampling, the received symbols are expressed as

(1) $$y=H_{t}x+n,$$

where y is the received vector of *M* channel hydrophones, where $y={\left[y_{1}^{T},y_{2}^{T},\ldots y_{K}^{T}\right]}^{T}$ with $y_{k}=\left[y_{1,k},y_{2,k},\ldots ,y_{M,k}\right]$, n is the additive noise vector where $n={\left[n_{1}^{T},n_{2}^{T},\ldots n_{K}^{T}\right]}^{T}$ with $n_{k}={\left[n_{1,k},n_{2,k},\ldots n_{M,K}\right]}^{T}$, and $n_{m,k}∼CN\left(0,\sigma_{n,m}^{2}\right)$ for ease of modeling, where CN(·) denotes the complex Gaussian distribution, and $\sigma_{n,m}^{2}$ is the variance of the noise. $H_{t}$ is the UWAC channel state and block circulant matrix with the first column ${\left[h_{0}^{T},h_{1}^{T},\ldots ,h_{L-1}^{T},0_{M,K-L}\right]}^{T}$, where $h_{l}=\left[h_{l,1},h_{l,2},\ldots ,h_{l,M}\right]$, and *L* is the channel length.

## 3. Proposed FDTE with Iterative Channel Estimation

Here, a SIMO IFDTE scheme with low complexity iterative channel estimation is proposed. First, the sparse channel estimation is designed. Second, the proposed IFDTE processes the received symbols in terms of the above estimated CSI. The combination of bidirectional extrinsic information is adopted to accelerate the equalization convergence. The a posteriori soft decision promotes the performance for channel estimation. Finally, the flow chart of the entire scheme with complexity analyses are presented to enhance understanding.

Traditional turbo equalization mainly exploits the MMSE criterion for independent Gaussian distribution (*i.i.d*) symbols. The distribution characteristics are usually ignored, easily resulting in equalization performance being degraded. An EP equalization based on simple Gaussian distribution is proposed to approximate the actual posterior distribution of the transmission symbol through moment matching, so this scheme is used to effectively improve the equalization.

**Figure 2:**
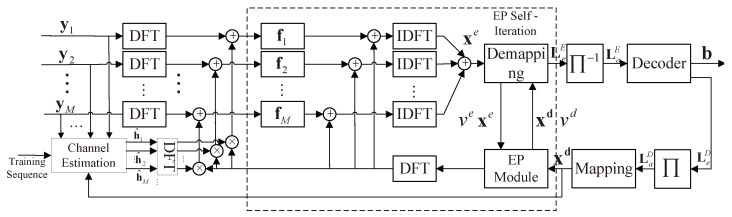
Proposed IFDTE with iterative channel estimation for the SIMO system in UWACs.

The entire turbo equalization can be described as follows. First, adaptive channel estimation is adopted to estimate the CSI in terms of training sequences and is fed back to the turbo equalization in the frequency domain. Second, the moment of the a posteriori distribution of the transmitted codeword is approximately matched through the frequency domain EP turbo equalization in terms of the channel state and a priori decoding information $L_{D}^{a}$. Finally, the a posteriori information calculated by the a posteriori probability is adopted after the self-iteration based on the EP. This information is used to calculate the extrinsic information $L_{E}^{e}$ of the equalization, and $L_{E}^{e}$ is fed back to the decoder. If the maximum turbo iteration is not reached, the $L_{D}^{e}$ of the decoder is outputted. Otherwise, the final results of the codeword is outputted. The block diagram of the FDTE with the EP in a SIMO UWAC system is designed and shown in Figure 2.

### 3.1. Sparse Adaptive Channel Estimation

Channel estimation is a key process in the equalization of received UWAC signals. The SZA-IPNLMS is adopted to estimate the sparse channel efficiently.

The length of training sequence $x_{ts}\left(k\right)$ at the *k*-th moment is *L*, and the training sequence is $x_{ts}\left(k\right)={\left[x_{ts,k},x_{ts,k-1},\ldots ,x_{ts,k-L+1}\right]}^{T}$. The received signal by the *m*-th hydrophone at the *k*-th moment is, and the offset of channel estimation is $e_{m}\left(k\right)=y_{m,k}-x_{ts}^{T}\left(k\right){\hat{h}}_{m}\left(k\right)$. ${\hat{h}}_{m}\left(k\right)={\left[{\hat{h}}_{m,k}^{0},{\hat{h}}_{m,k}^{1},\ldots ,{\hat{h}}_{m,k}^{L-1}\right]}^{T}$ represents the estimated value of the impulse response of the UWAC channel at the k-th moment, and the channel coefficients of $\hat{h_{m}\left(k+1\right)}$ are updated as

(2) $$\begin{array}{c} {\hat{h}}_{m}\left(k+1\right)=\mu \frac{e_{m}^{*}\left(k\right)\Theta_{m,k}x_{ts}\left(k\right)}{x_{ts}{\left(k\right)}^{T}\Theta_{m,k}x_{ts}\left(k\right)+\delta }+{\hat{h}}_{m}\left(k\right)-Q_{m,k}{\bar{h}}_{m}\left(k\right), \end{array}$$

where δ is the adjustment factor to avoid the stopping of the iteration due to the extremely small denominator in the initial stage. $\Theta_{m,k}=diag\left(\theta_{m,k}^{0},\theta_{m,k}^{1},\ldots ,\theta_{m,k}^{L-1}\right)$ is a diagonal proportionate matrix, $\theta_{m,k}^{l}$ is calculated as

(3) $$\theta_{m,k}^{l}=\frac{1-\alpha }{2L}+\frac{\left(1+\alpha \right)\left|{\hat{h}}_{m,k}^{l}\right|}{2\left(∥{\hat{h}}_{m}\left(k\right)∥\right)+\varepsilon },l=0,\cdots ,L-1,$$

where |·| represents absolute value operation, ||·|| represents $l_{1}$ norm, and $Q_{m,k}$ indicates the norm constraint factor and is expressed as

(4) $$Q_{m,k}=\left\{\begin{array}{c} \gamma ,\begin{array}{c} \frac{|{\left[{\hat{h}}_{m}\left(k\right)\right]}_{p}|}{\|{\hat{h}}_{m}\left(k\right){\|}_{\infty }} \end{array}<\beta \\ 0,\begin{array}{c} \begin{array}{c} \frac{|{\left[{\hat{h}}_{m}\left(k\right)\right]}_{p}|}{\|{\hat{h}}_{m}\left(k\right){\|}_{\infty }}\geq \beta \end{array} \end{array} \end{array},\right.$$

where β is the threshold, γ is shrinkage step size, ${\left[\cdot \right]}_{p}$ represents the *p*-th element of the vector, and ${\bar{h}}_{m}\left(k\right)$ is given by

(5) $${\left[{\bar{h}}_{m}\left(k\right)\right]}_{p}=\left\{\begin{array}{c} \frac{{\left[{\bar{h}}_{m}\left(k\right)\right]}_{p}}{|{\left[{\bar{h}}_{m}\left(k\right)\right]}_{p}|},\begin{array}{c} {\left[{\bar{h}}_{m}\left(k\right)\right]}_{p}\neq 0 \end{array}\\ 0,{\left[{\bar{h}}_{m}\left(k\right)\right]}_{p}=0 \end{array}\right..$$

The noise variance is estimated using the equation in [16]:

(6) $${\hat{\sigma }}_{n,m}^{2}=\mu e_{m}\left(k\right)e_{m}^{*}\left(k\right)+\left(1-\mu \right){\hat{\sigma }}_{n,m}^{2},$$

In the initial iteration, ${\hat{\sigma }}_{n,m}^{2}=0$. The channel estimation based on soft feedback from the turbo equalization is performed, and the offset of the soft iterative channel estimation is

(7) $$d_{m}\left(k\right)=y_{m,k}-{{\hat{h}}_{m}}^{T}\left(k\right)\bar{x}\left(k\right),$$

where $\bar{x}\left(k\right)={\left[{\bar{x}}_{k},{\bar{x}}_{k-1},\ldots ,{\bar{x}}_{k-L+1}\right]}^{T}$, ${\bar{x}}_{k}$ can be obtained by

(8) $${\bar{x}}_{k}=\sum_{q=1}^{Q}\alpha_{q}\prod_{q=1}^{Q}(1+\left(1-2c_{k,q}\right)\tanh\left(L_{a}^{D}\left(c_{k,q}\right)/2\right)/2).$$

The extrinsic information $L_{a}^{D}\left(c_{k,q}\right)$ fits the Gaussian distribution. Due to the estimation offset of $v_{k}$ existing between the soft mapping and the actual symbols, the relationship between them is denoted as

(9) $${\bar{x}}_{k}=x_{k}+v_{k}.$$

Therefore, the offset of channel estimation needs to be subtracted to update the noise variance estimation in each iteration, and the variance of the soft decision is given by

(10) $${\hat{\sigma }}_{n,m}^{2}=\mu \left(d_{m}\left(k\right)d_{m}^{*}\left(k\right)-h_{m}^{T}\left(k-1\right)V_{k}h_{m}^{*}\left(k-1\right)\right)+\left(1-\mu \right){\hat{\sigma }}_{n,m}^{2},$$

where $V_{k}$ is the covariance matrix of $x_{ts}\left(k\right)$. When iterative channel estimation is performed, the channel coefficients are updated by the DIPNLMS [21], and the update of channel coefficients is denoted as

(11) $$h_{m}\left(k+1\right)=\left\{\begin{array}{c} \left(1-\frac{\gamma_{t}}{\left|d(k)\right|}\right)\frac{d^{*}\left(k\right)\Theta_{m,k}^{D}\bar{x}\left(k\right)}{\bar{x}{\left(k\right)}^{T}\Theta_{m,k}^{D}\bar{x}\left(k\right)+\delta }+\\ h_{m}\left(k\right)-Q_{m,k}{\bar{h}}_{m}\left(k\right),\left|d(k)\right|>\gamma_{t}\\ h_{m}\left(k\right)\;\;\mathrm{otherwise}.\; \end{array},\right.$$

where $\gamma_{t}$ is the defined membership, and $\gamma_{t}=\sqrt{2\sigma_{n,m}^{2}}$ is the differential proportionate diagonal matrix. By updating the step size matrix by period D, $\theta_{m,k}^{l}$ of $\Theta_{m,k}^{D}$ is calculated as

(12) $$\theta_{m,k}^{l}=\alpha +\frac{1}{D}\sum_{i=1}^{D}\frac{\left(1-\alpha \right)|h_{l,D+i}-h_{l,D}|}{\frac{1}{L}\sum_{j=0}^{L-1}|h_{j,D+i}-h_{j,D}|+\delta },$$

when k≤D, the proportionate matrix $\Theta_{m,k}^{D}$ is calculated with the channel estimation by reusing the training sequence.

### 3.2. Expectation Propagation Interference Cancellation FDTE

The frequency domain expression of the m-th hydrophone received symbols in the m-th hydrophone is expressed as

(13) $$Y_{m}=H_{f,m}X_{m}+N_{m},$$

where $Y_{m}=Fy_{m}$, $y_{m}={\left[y_{1,m},y_{2,m},\ldots ,y_{K,m}\right]}^{T}$, $H_{f,m}=F^{H}H_{t,m}F$ is the circulant matrix, the first column of $H_{t,m}$ is ${\left[h_{0,m},h_{1,m},\ldots ,h_{L-1,m},0_{K-L}\right]}^{T}$, $X_{m}=Fx_{m}$, where $x_{m}={\left[x_{1,m},x_{2,m},\ldots ,x_{K,m}\right]}^{T}$, and F is the normalized *K*-discrete Fourier transformation (DFT) matrix. Thus, $H_{f,m}=diag\left(H_{f,m,1},H_{f,m,2},\ldots ,H_{f,m,K}\right)$, where diag(·) is the operation of diagonalization. $N_{m}$ is obtained through DFT from $n_{m}={\left[n_{1,m},n_{2,m},\ldots ,n_{K,m}\right]}^{T}$.

In Figure 2, the scheme mainly includes a symbol probability estimation module based on the EP and frequency domain equalization. In accordance with the extrinsic information from the decoder, the a priori probability is calculated as

(14) $$P\left(x_{k}\right)=\prod_{q=1}^{Q}(1+\left(1-2c_{k,q}\right)\tanh\left(L_{D}^{e}\left(c_{k,q}\right)/2\right)/2,$$

where $L_{D}^{e}\left(c_{k,q}\right)$ is the extrinsic information from the decoder and serves as the a posteriori information in equalization. In the first iteration, no a posteriori information input is found, and $L_{D}^{e}\left(c_{k,q}\right)$ is initially set to zero. On the basis of the Bayesian principle, the discrete a posteriori probability can be calculated using $P({\bar{x}}_{k}|x_{k})$ and a posteriori probability $P(x_{k})$. With the initial iteration, $x_{k}^{e}=0$, and $v^{e}=10^{12}$. The a posteriori probability $P(x_{k}=\alpha_{q}|{\bar{x}}_{k})$ is then derived and expressed as

(15) $$P\left(x_{k}=\alpha_{q}|{\bar{x}}_{k}\right)\propto \exp\left(-|\alpha_{q}-x_{k}^{e}{|}^{2}/v^{e}\right)P_{k}\left(\alpha_{q}\right),\forall \alpha_{q}\in A.$$

The calculation of (15) is dependent on the two assumptions below. The a posteriori distribution fits the Gaussian distribution, and $P(x_{k}=\alpha_{q}|{\bar{x}}_{k})$ is regarded as the a posteriori approximation factor of the EP. Moment matching is adopted to approximate the a posteriori distribution iteratively. The mean $\mu_{k}^{d}$ and variance γ of the distribution are calculated by

(16) $$\mu_{k}^{d}=E_{p(x_{k}=\alpha_{q}|{\bar{x}}_{k})}\left[x_{k}\right]=\sum_{\alpha_{q}\in A}\alpha_{q}P(x_{k}=\alpha_{q}|{\bar{x}}_{k}),$$

(17) $$\begin{array}{cc} \gamma^{d}=E\left(Var_{p(x_{k}|{\bar{x}}_{k})}\left[x_{k}\right]\right) & =E(\sum_{\alpha_{q}\in A}{|\alpha_{q}|}^{2}P\left(x_{k}|{\bar{x}}_{k}\right))\\ & =E(1-|\mu_{k}^{d}{|}^{2}). \end{array}$$

The mean $x_{k}^{d}$ and variance $v^{d}$ of the a posteriori distribution are estimated with moment matching. In accordance with the Bayesian principle and Gaussian operation, mean $x_{k}^{d(next)}$ and variance $v^{d(next)}$ are presented as

(18) $$v^{d(next)}={\left(\left(1-\beta_{d}\right)\frac{v^{e}\gamma^{d}}{v^{e}-\gamma^{d}}+\beta {}_{d}v^{d(prev)}\right)}^{-1},$$

(19) $$x_{k}^{d(next)}=\left(1-\beta_{d}\right)\frac{\mu_{k}^{d}v^{e}-x_{k}^{e}\gamma^{d}}{v^{e}-\gamma^{d}}+\beta_{d}x_{k}^{d(prev)}.$$

The negative variance can be effectively avoided by setting the damping factor $\beta_{d}$, and the stability of the system can be ensured by controlling the update step size. ${\left(\cdot \right)}^{\left(next\right)}$ and ${\left(\cdot \right)}^{\left(prev\right)}$ represent the next and previous states, respectively. $x_{k}^{d(prev)}=0$ and $v^{d(prev)}=1$ because no previous state exists in the initial iteration. In conclusion, the above procedures are the refining process of the a priori information in equalization.

Subsequently, the optimized a posteriori information and CSI are exploited to process the received signal. The a posteriori probability of the received symbols fits the Gaussian distribution [10]. The MMSE criterion is used to calculate and obtain the variance $\gamma^{e}$ and mean ${\bar{X}}_{k}$ of the a posteriori distribution [15]:

(20) $$\gamma^{e}=v^{d}\left(1-v^{d}\xi \right),$$

(21) $$\begin{array}{c} {\bar{X}}_{k}=X_{k}^{d}+v^{d}\xi \sum_{m=1}^{M}f_{m,k}^{*}\left(Y_{m,k}-H_{f,m,k}^{H}X_{k}^{d}\right), \end{array}$$

where $X^{d}={\left[X_{1}^{d},X_{2}^{d},\ldots ,X_{K}^{d}\right]}^{T}$ is obtained with the DFT of $x^{d}$, and $\bar{X}={\left[{\bar{X}}_{1},{\bar{X}}_{2},\ldots ,{\bar{X}}_{K}\right]}^{T}$ is the equalized sequence of symbols in the frequency domain. $\bar{x}={\left[{\bar{x}}_{1},{\bar{x}}_{2},\ldots ,{\bar{x}}_{K}\right]}^{T}$ is obtained with the inverse DFT (IDFT) of $\bar{X}$. The filter coefficient $f_{m,k}$ and ξ are represented as

(22) $$f_{m,k}=\xi^{-1}H_{f,m,k}/\left({\hat{\sigma }}_{n,m}^{2}+v^{d}|H_{f,m,k}{|}^{2}\right),$$

(23) $$\xi =K^{-1}\sum_{k=1}^{K}{|H_{f,m,k}|}^{2}/\left({\hat{\sigma }}_{n,m}^{2}+v^{d}|H_{f,m,k}{|}^{2}\right),$$

The mean and variance of the estimated a posteriori distribution are derived by (18)–(21). The marginal distribution $p({\bar{x}}_{k}|x_{k})$ is estimated through the EP [16,17], and the mean and variance of $p({\bar{x}}_{k}|x_{k})$ are given by

(24) $$\begin{array}{cc} X_{k}^{e}=\frac{{\bar{X}}_{k}v^{d}-X_{k}^{d}\gamma^{e}}{v^{d}-\gamma^{e}} & =X_{k}^{d}+\sum_{k=1}^{M}f_{m,k}^{*}\left(Y_{m,k}-H_{f,m,k}X_{k}^{d}\right), \end{array}$$

(25) $$v^{e}=\frac{v^{d}\gamma^{e}}{v^{d}-\gamma^{e}}=\xi^{-1}-v^{d},$$

where $X^{e}=\left[X_{1}^{e},X_{2}^{e},\ldots ,X_{K}^{e}\right]$, and $x^{e}=\left[x_{1}^{e},x_{2}^{e},\ldots ,x_{K}^{e}\right]$ is obtained with the IDFT of $X^{e}$. Subsequently, the estimated mean and variance of the marginal distribution obtained by (24) and (25) are fed back to (15), and the EP is executed until the maximum EP self-iteration is reached. On the basis of the estimated a posteriori probability of the transmitted symbols and the Bayesian principle, the extrinsic information $L_{E}^{e}\left(c_{k,q}\right)$ is calculated as

(26) $$L_{E}^{e}\left(c_{k,q}\right)=\ln\frac{\sum_{\alpha_{q}\in A_{q}^{0}}q_{k}\left(\alpha_{q}\right)}{\sum_{\alpha_{q}\in A_{q}^{1}}q_{k}\left(\alpha_{q}\right)}-L_{D}^{a}\left(c_{k,q}\right),$$

where $c_{k,q}$ represents the *k*-th modulation symbol of the *q*-th code-word, and $A_{q}^{0}$ and $A_{q}^{1}$ are the sets of the *q*-th code word of the modulation symbols of “0” and “1”, respectively. The extrinsic information $L_{d}^{a}$ obtained in the equalization is de-interleaved and inputted into the Log-MAP decoder. $L_{d}^{a}$ is outputted in terms of the Log-MAP criterion. With the maximum turbo iterations reached, code word $\hat{b_{i}}$ decoded by the decoder is expressed as

(27) $${\hat{b}}_{i}=\underset{b\in \{0,1\}}{\arg\;\max}\;P\left(b_{i}=b|L\left(b_{1}\right),\ldots ,L\left(b_{N_{b}}\right)\right).$$

### 3.3. Bidirectional Combination

The combination of extrinsic information for the bidirectional turbo equalization mainly includes the two following methods, namely a mean combining scheme [27] and a joint Gaussian scheme [28]. The IFDTE and reversal IFDTE process the same received signal of the same channel only in a time-reversed order, and the IFDTE uses Gaussian distribution to approximate the true a posteriori distribution. The joint extrinsic information is treated to fit the joint Gaussian distribution [29]. The bidirectional structure has two equalizations, namely forward and reversal equalizations [30]. The combined extrinsic information fits the joint Gaussian distribution and is expressed as

(28) $$\begin{array}{cc} \; & P\left(L_{e}^{E}\left(c_{k,j}\right),{\tilde{L}}_{e}^{E}\left(c_{k,j}\right)|c_{k,j}\right)\\ & =\frac{1}{2\pi \sqrt{\det(\Phi )}}\exp\left\{-\frac{1}{2}{\left(L_{k}-\mu_{k}\right)}^{T}\Phi^{-1}\left(L_{k}-\mu_{k}\right)\right\}\\ & =\frac{1}{2\pi \sigma_{1}\sigma_{2}\sqrt{1-\rho^{2}}}\exp\left\{\frac{A_{1}^{2}\left(\pm 1\right)+2\rho^{2}A_{1}\left(\pm 1\right)A_{2}\left(\pm 1\right)-A_{2}^{2}\left(\pm 1\right)}{2\left(1-\rho^{2}\right)}\right\}, \end{array}$$

(29) $$\begin{array}{c} \Phi =\left[\begin{array}{cc} \begin{array}{c} {\left(\sigma_{1}\right)}^{2}\\ \rho \sigma_{1}\sigma_{2} \end{array} & \begin{array}{c} \rho \sigma_{1}\sigma_{2}\\ {\left(\sigma_{2}\right)}^{2} \end{array} \end{array}\right],¯k=\alpha_{q}\left[\gamma_{1},\gamma_{2}\right],\\ A_{1}\left(\pm 1\right)=\frac{\left(L_{E}^{e}\left(c_{k,q}\right)\mp \gamma_{1}\right)}{\sigma_{1}},A_{2}\left(\pm 1\right)=\frac{\left({\tilde{L}}_{E}^{e}\left(c_{k,q}\right)\mp \gamma_{2}\right)}{\sigma_{2}}, \end{array}$$

where $L_{k}=\left[L_{E}^{e}\left(c_{k,q}\right),{\tilde{L}}_{E}^{e}\left(c_{k,q}\right)\right]$, $L_{E}^{e}\left(c_{k,q}\right)$ represents the forward extrinsic information, and ${\tilde{L}}_{E}^{e}\left(c_{k,q}\right)$ represents the reversal extrinsic information through a time-reversal operation. $\gamma_{1}$, $\sigma_{1}^{2}$ are the mean and variance of $L_{e}^{E}$, $\gamma_{2}$, $\sigma_{2}^{2}$ are the mean and variance of ${\tilde{L}}_{E}^{e}$, and ρ is the correlation coefficient of forward and reversal extrinsic information and is given by

(30) $$\rho =\frac{\sum_{k=1}^{K}\left[L_{E}^{e}\left(c_{k,q}\right)-\gamma_{1}\right]\left[{\tilde{L}}_{E}^{e}\left(c_{k,q}\right)-\gamma_{2}\right]}{\left(K-1\right)\sigma_{1}\sigma_{2}}.$$

Given the probability distribution of the extrinsic information, $L_{E}\left(c_{k,q}\right)$ is calculated as

(31) $$L_{E}\left(c_{k,j}\right)≜\log\frac{P\left(L_{E}^{e}\left(c_{k,j}\right),{\tilde{L}}_{E}^{e}\left(c_{k,j}\right)|c_{k,j}=0\right)}{P\left(L_{E}^{e}\left(c_{k,j}\right),{\tilde{L}}_{E}^{e}\left(c_{k,j}\right)|c_{k,j}=1\right)}.$$

The combined extrinsic information $L_{E}(c_{k,q})$ by inputting (28) into (31) is expressed as

(32) $$L_{E}\left(c_{k,q}\right)=\lambda_{1}L_{E}^{e}\left(c_{k,q}\right)+\lambda_{2}{\tilde{L}}_{E}^{e}\left(c_{k,q}\right),$$

where $\lambda_{1}=\frac{2/\sigma_{1}}{1-\rho^{2}}\left(\frac{\gamma_{1}}{\sigma_{1}}-\frac{\rho \gamma_{2}}{\sigma_{2}}\right)$, $\lambda_{2}=\frac{2/\sigma_{2}}{1-\rho }\left(\frac{\gamma_{2}}{\sigma_{2}}-\frac{\rho \gamma_{1}}{\sigma_{1}}\right)$, and the parameters of the forward and reversal equalization can be equivalent due to the same equalized symbols. Thus, $\gamma_{1}\approx \gamma_{2}$, and $\sigma_{1}\approx \sigma_{2}$. $\sigma_{1}\approx \sqrt{2\gamma_{1}}$, due to phase-shift modulation and the combined extrinsic information, can be rewritten as

(33) $$L_{E}\left(c_{k,q}\right)=\frac{1}{1+\rho }\left(L_{E}^{e}\left(c_{k,q}\right)+{\tilde{L}}_{E}^{e}\left(c_{k,q}\right)\right).$$

### 3.4. A Posteriori Soft Decision for Iterative Channel Estimation

After the first iteration, the symbol of the a posteriori soft decision ${\overset{ˇ}{x}}_{k}$ of equalized symbol $x_{k}^{e}$ can be obtained, similar to those in [9,14]:

(34) $${\overset{ˇ}{x}}_{k}=\sum_{\alpha_{q}\in A}\alpha_{q}P\left(x_{k}=\alpha_{q}|x_{k}^{e}\right),$$

where the a posteriori probability $P\left(x_{k}=\alpha_{q}|{\hat{x}}_{k}^{e}\right)$ is given by

(35) $$P\left(x_{k}=\alpha_{q}|x_{k}^{e}\right)=\frac{P\left(x_{k}^{e}|x_{k}=\alpha_{q}\right)P\left(x_{k}=\alpha_{q}\right)}{P\left(x_{k}^{e}\right)}.$$

In (32), $P(x_{k})$ is calculated in (7), and $P(x_{k}^{e})$ is the normalization factor. The marginal $P(x_{k}^{e}|x_{k}=\alpha_{q})$ is calculated as

(36) $$P\left(x_{k}^{e}|x_{k}=\alpha_{q}\right)\propto \exp\left(-|x_{k}^{e}-\alpha_{q}{|}^{2}/v^{e}\right).$$

With the continuous iteration of the turbo equalization, the reliability of the a posteriori soft feedback keeps increasing to accelerate the convergence of the estimation.

Thus, the iterative channel estimation in (9) after the first iteration can be rewritten as

(37) $$h_{m}\left(k+1\right)=\left\{\begin{array}{c} \left(1-\frac{\gamma }{\left|{\overset{ˇ}{d}}_{m}\left(k\right)\right|}\right)\frac{{\overset{ˇ}{d}}_{m}^{*}\left(k\right)\Theta_{m}^{D}\overset{ˇ}{x}\left(k\right)}{\overset{ˇ}{x}{\left(k\right)}^{T}\Theta_{m}^{D}\overset{ˇ}{x}\left(k\right)+\delta }+\\ h_{m}\left(k\right)-Q_{m,k}{\bar{h}}_{m}\left(k\right),\left|\overset{ˇ}{d}\left(k\right)\right|>\gamma \\ h_{m}\left(k\right),\;\;\mathrm{otherwise}.\; \end{array}\right.,$$

where ${\overset{ˇ}{d}}_{m}\left(k\right)=y_{m}\left(k\right)-{{\hat{h}}_{m}}^{T}\left(k\right)\overset{ˇ}{x}\left(k\right)$ with ${\overset{ˇ}{x}}_{k}=\left[{\overset{ˇ}{x}}_{1},{\overset{ˇ}{x}}_{2},\ldots ,{\overset{ˇ}{x}}_{K}\right]$.

### 3.5. Summary of the Proposed Algorithm

**Figure 3:**
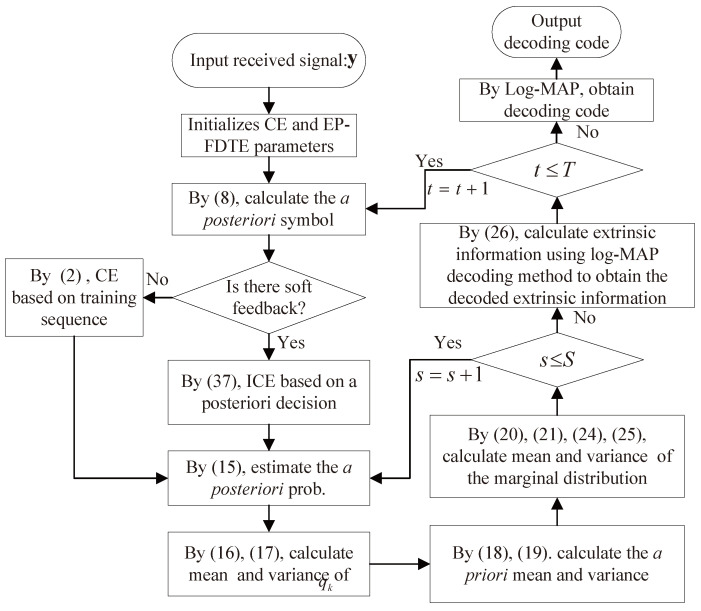
Flow chart of the IFDTE with iterative channel estimation.

The proposed algorithm mainly includes three parts, namely low complexity iterative sparse channel estimation, the IFDTE, and the decoder. The flow chart of the proposed scheme is shown in Figure 3 and depicted in Table 1.

### 3.6. Analysis of the Computational Complexity

In this section, the complexity analysis and a comparison for the different turbo equalizations are provided. The complexity comes from four parts, namely the equalization filtering, symbol estimation, computation of the conditional mean, and variance [9]. The main complexity is generally from equalization filtering. In the proposed IFDTE, the parallel block equalization filtering is made using FFT with significant efficient parallel convolution computation, thus improving the computational complexity significantly by reducing many multiplications in the filtering calculation of the TDTE. However, through the parallel implementation of the proposed block equalization filter, the proposed scheme occupies much more memory usage than the parallel FFT calculation, other than for the serial convolutional one, for the cost of fast computation. And the additional memory usage is mainly related to the degree of parallelism of the main convolutional filtering. In addition, the symbol estimation also occupies some complexity in the proposed IFDTE and it is moderate, as shown in Table 2. Then, the complexity comparison can be analyzed as follows. Without a loss of generality, the computation complexity in terms of complex multiplication is adopted. Suppose the number of receiving hydrophones is *M*, the maximum number of the EP self-iteration is *S*, the length of FFT is *K*, the length of the feedforward filter is $N_{1}$, the length of the feedbackward filter is $N_{2}$, the total length of the filter for the TDTE is $N_{s}=N_{1}+N_{2}+1$, and the length of the UWAC channel is *L*.

From Table 2, the complexity of the proposed IFDTE is approximately *S* times higher than the FDDF-FDTE. This result is due to the self-iteration based on the EP in estimating the actual characteristic of the a posteriori distribution. However, the complexity of TDTE is proportional to the length of the filter and the UWAC channel. The UWAC channels are longer than 50 taps, the length of equalization is longer than that of channels [2], and the number of self-iterations is smaller than 10. Therefore, the computational complexity of the LE and SDFE is larger than that of the IFDTE.

## 4. Simulation Results and Analyses

Simulations are conducted to illustrate the performance of the proposed scheme. The data bits are encoded with a 1/2 rate binary convolutional code with generator polynomial [171, 133], and the length of the generated coded bits is 2048. The coded bits are then interleaved with a random interleaver and modulated with QPSK. The training sequence with a length of 300 is inserted into the front of blocks, the total number of blocks is 100, and the total repetitions of the Monte Carlo simulation is 100. First, the CSI in the receiver is estimated through sparse iterative channel estimation. Second, the IFDTE processes the received signals with five self-iterations, and the damping factor $\beta_{d}$ is set as $0.7\times 0.9^{s}$ [13], where *s* is the *s*-th time of self-iteration. Finally, the log-domain maximum a posteriori probability (Log-MAP) is used for the decoder. An additive white Gaussian noise with *i.i.d.* zero-mean real and imaginary components is used in the simulations and is completely described by its variance.

### 4.1. Experimental Environment

The UWAC channels are generated using the acoustic channel simulator in reference [21] to approximate the practical channel. We adopt the environmental settings for two experiments, namely the Surface Processes Acoustic Communications Experiment, conducted in 2008 (SPACE’08) [21], and the Mobile Acoustic Communications Experiment conducted in 2010 (MACE’10) [31]. The simulation parameters are mainly chosen from the above two channel models of the SPACE’08 and MACE’10 experiment settings, and other individual parameters in our simulations are listed in Table 3. Thus, they equivalently provide enough information for the channel verification of the channel models, noise characteristics, and so on, together. The acoustic channel simulator adopts a statistical channel model to incorporate physical laws of acoustic propagation (frequency-dependent attenuation, bottom/surface reflections) and the effects of inevitable random local displacements. Additionally, random displacements on two scales are employed in the acoustic channel simulator as small- and large-scale effects, with the distances on the order of a few wavelengths and many wavelengths, respectively. In summary, the main system setting of parameters is shown in Table 3 and they are responsible for the completeness of our experiment simulations plus the two above channel models.

**Figure 4:**
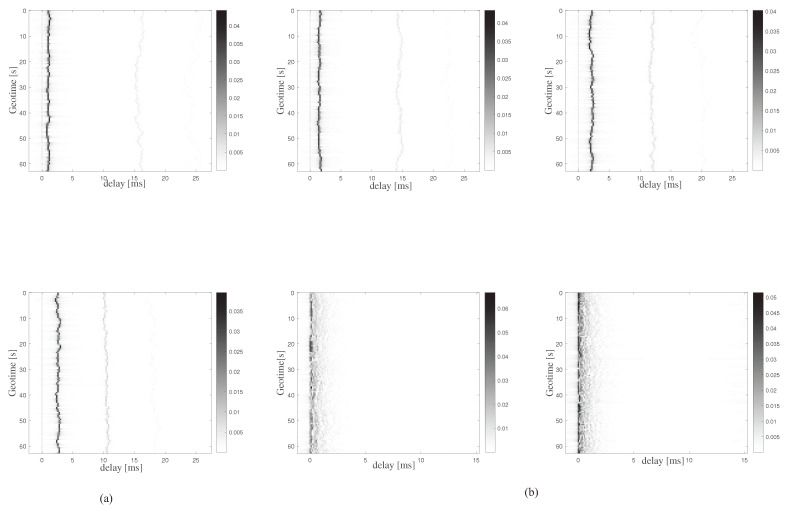
Time evolution of the magnitude impulse response for (**a**) Space’08 and (**b**) MACE’10.

Figure 4 shows the simulated time-varying UWAC channels. Figure 4a,b show the ensembles of the simulated channel impulse response based on the SPACE’08 and MACE’10 experiment settings over a duration of 1 min. Figure 4a corresponds to the receiver with depths of 2 m and 6 m, respectively. Figure 4b corresponds to the receiver with depths of 20 m, 30 m, 40 m, and 50 m, respectively.

### 4.2. Comparison between Different Channel Estimation

**Figure 5:**
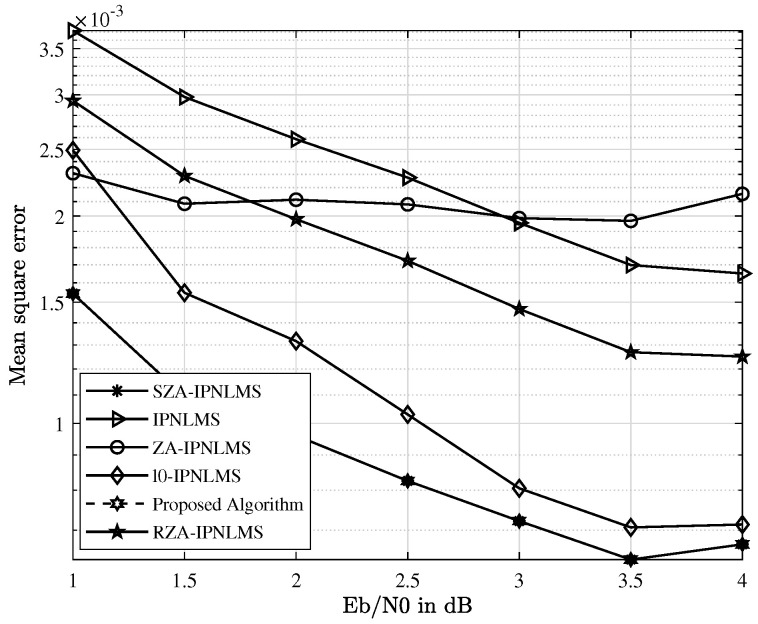
MSE comparison with different channel estimations in the UWAC channel.

**Figure 6:**
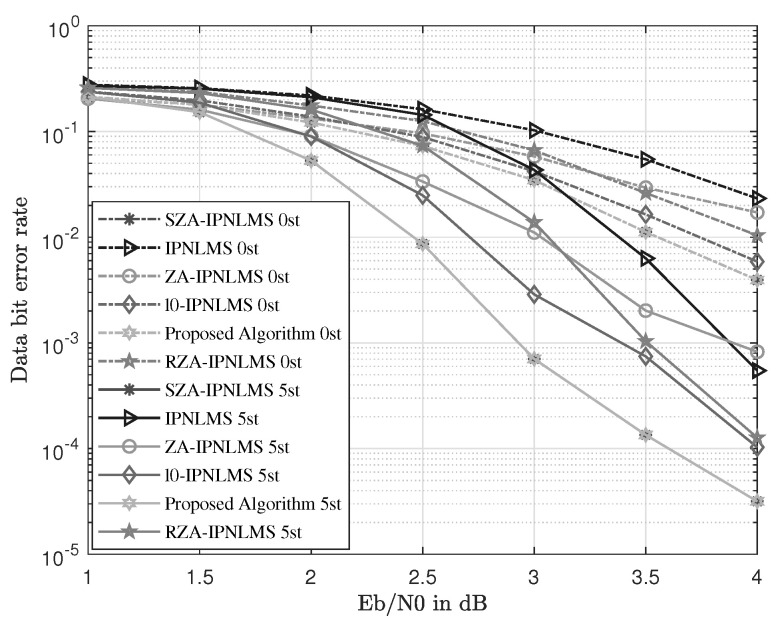
BER comparison with different channel estimations in the UWAC channel.

This subsection mainly compares the performance of the proposed scheme with other channel estimations. The UWAC channel used in this section is the first channel in Figure 4b. The IPNLMS, ZA-IPNLMS, RZA-IPNLMS, $l_{0}$-IPNLMS, SZA-IPNLMS, and a hybrid channel estimation are compared. The proposed channel estimation is made up of the SZA-IPNLMS and SM SZA-DIPNLMS. The common parameters of channel estimation for all schemes are set as step size μ=0.2, adjustment factor α=0, and regular factor δ=0.01. The norm constraint parameter of the ZA-IPNLMS, RZA-IPNLMS, and $l_{0}$-IPNLMS is $\kappa =5\times 10^{-6}$, and the one for the SZA-IPNLMS and SM SZA-DIPNLMS, is set as $\kappa =6\times 10^{-5}$. The hard threshold for $l_{0}$-IPNLMS is set as β=10, the proportionate threshold of the SZA-IPNLMS and SM SZA-DIPNLMS is set as β=0.1, and the update period of SM SZA-DIPNLMS is *L*/4. The norm constraint parameter and the proportionate threshold of the proposed IFDTE scheme are configured identically to those of the SZA-IPNLMS and SM SZA-DIPNLMS. Thus, it equivalently provides enough information for the simulation of the proposed IFDTE scheme for performance verification. Finally, the curves of the MSE and BER are simulated and shown in Figure 5 and Figure 6.

In Figure 5, the MSE performance for different channel estimations is presented. Under the same SNR, both the SZA-IPNLMS and the proposed algorithm have a lower MSE than those of other channel estimation schemes and they are almost overlapped together. MSE is the exact performance indication for the equalization since it illustrates the difference between the estimated values and the true ones. With the increase in SNR to 3.5 dB, the floor of the SZA-IPNLMS is lower than other channel estimations. Different from the traditional hard threshold, the SZA-IPNLMS gives a dynamical norm penalty term in terms of the ratio of channel coefficients to the maximum one in (4) and (5). The performance of the SZA-IPNLMS is better as the small coefficients of the UWAC channel estimate precisely.

In Figure 6, the BER curves of the same equalization with different channel estimations are presented. The proposed IFDTE scheme obtains the same and best BER performance as those of the SZA-IPNLMS among all schemes, where it obtains a BER of $7.15\times 10^{-4}$, $1.35\times 10^{-4}$ and $3.65\times 10^{-5}$ at SNR of 3 dB, 3.5 dB, and 4 dB, respectively. The BER performance for the equalization with the SZA-IPNLMS under the same SNR is better than others because the SZA-IPNLMS provides more precise CSI. Under the same BER, BER = $1\times 10^{-3}$, and the SZA-IPNLMS has 1.9 and 0.5 dB compared with the IPNLMS and $l_{0}$-IPNLMS, respectively. Thus, utilizing the SZA-IPNLMS effectively promotes the performance of the equalization.

**Figure 7:**
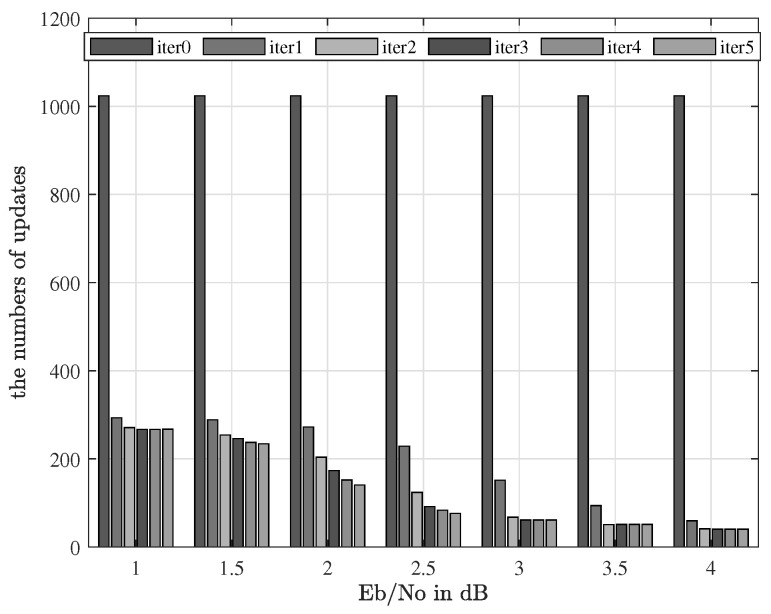
A histogram of the numbers of updates for channel coefficients.

In Figure 5 and Figure 6, the curve of the proposed scheme is in accordance with the one of the SZA-IPNLMS. Here, the mean square error curve of the proposed algorithm is identical to that of the best SZA-IPNLMS scheme and the proposed algorithm obtains the best performance among these schemes. Thus, the proposed scheme has minimal influence on the performance of the SZA-IPNLMS. In Figure 7, the numbers of updates for channel coefficients is reduced intensively, where the abscissa and ordinate correspond to the SNR and the number of updates, respectively. At the SNR of 1 dB, the numbers of updates are reduced by four-fifths. With SNR increasing, the numbers of updates for the channel coefficients see a downward trend. Therefore, the computational complexity is effectively reduced, which is quite suited for practice.

### 4.3. Comparison between Different Equalizations

**Figure 8:**
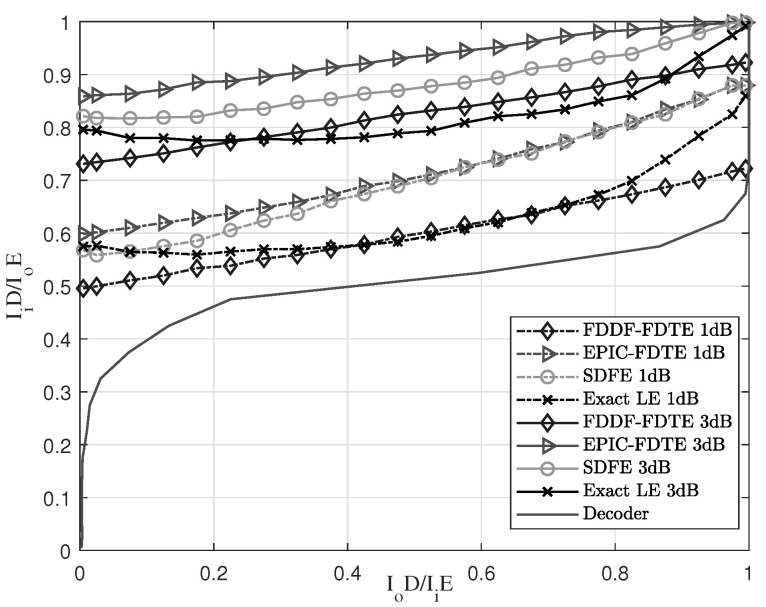
EXIT chart with different equalization schemes in the environment of SPACE’08.

**Figure 9:**
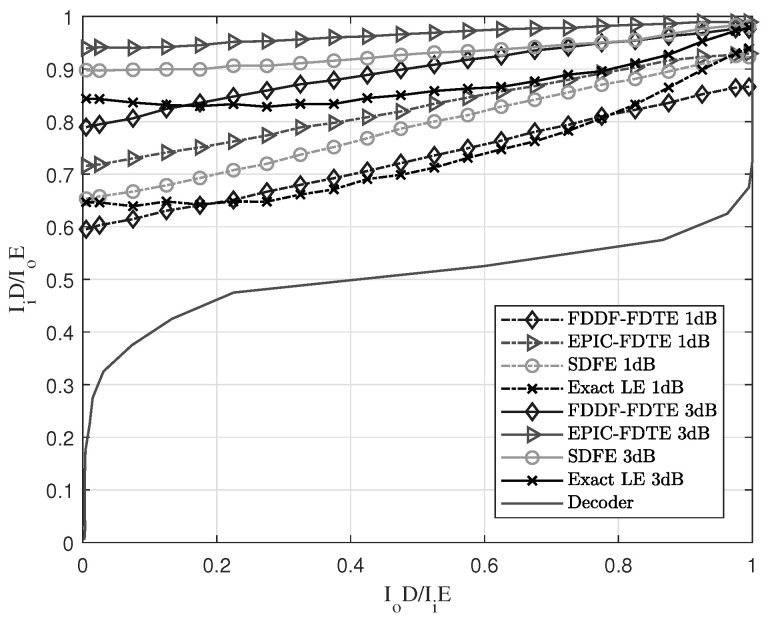
EXIT chart with different equalization schemes in the environment of MACE’10.

An EXIT chart [32] is adopted to analyze the convergence of the turbo equalization under the four-channel underwater acoustic channel. It is a powerful semi-analytical tool to analyse and design iteratively decoded systems with soft information exchange. The exchange of extrinsic information between the constituent decoders is verified using EXIT charts, which characterise the flow of entrinsic information exchange between the constituent decoders of a concatenated structure, such as the turbo equalization. It determines the order of soft-information exchange among the three components of the two constituent decoders and equalizer. Furthermore, it also exhibits beneficial decoding convergence after a fixed number of iterations, which resulted in a complexity reduction. The SNR is set to 1 and 3 dB, and the setting of other parameters is set the same as the above subsection of the comparison of channel estimation. The channel estimation adopts the SZA-IPNLMS in the initial stage and the SM SZA-DIPNLMS in the main stages of iterative channel estimation. The length of the forward filter for the SDFE and LE is set to 148, and the length of the feedback filter is set to 74. After numerical simulations, the results of the EXIT charts of different equalization schemes in SPACE’08 and MACE’10 are shown in Figure 8 and Figure 9, respectively.

In the EXIT chart, $I_{i}^{E}$ and $I_{o}^{E}$ denote the input and output mutual information of the equalization, and $I_{i}^{D}$ and $I_{o}^{D}$ denote those of the decoder. Information theory states that a larger amount of mutual information leads to a more reliable system [33]. Figure 8 and Figure 9 are in accordance with the analyses of EXIT charts in SPACE’08 and MACE’10. At 1 or 3 dB, the IFDTE is better than other equalization schemes in terms of early iteration due to the self-iteration of the EP. It has better performance under low SNR and it also has a wide “tunnel” area, and the width of “tunnel” is proportional to $I_{o}^{D}$. Thus, the wider the width is, the better the performance. Thus, the performance is ranked as IFDTE > SDFE > Exact-LE > FDDF-FDTE under 1 dB and IFDTE > SDFE > FDDF-FDTE > Exact-LE under 3 dB. Therefore, the IFDTE has a faster convergence and better performance than other equalization schemes.

**Figure 10:**
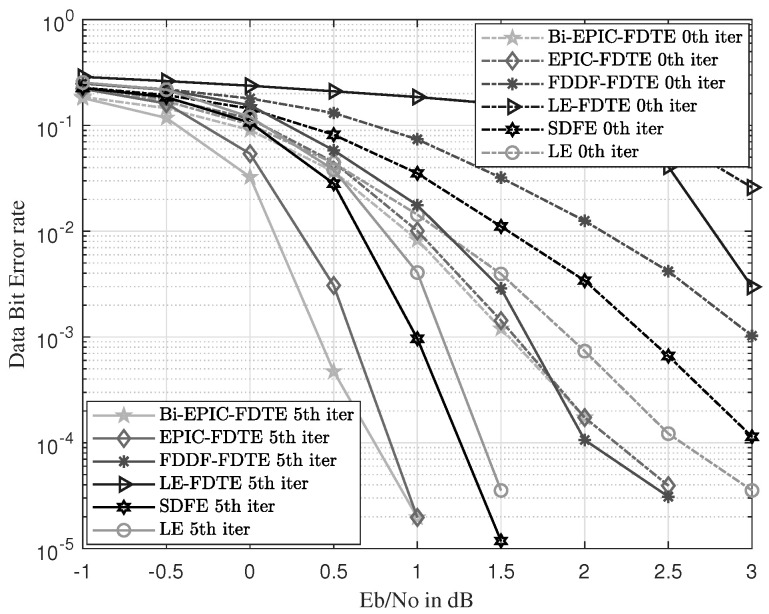
BER comparison with different equalizations in the environment of SPACE’08.

**Figure 11:**
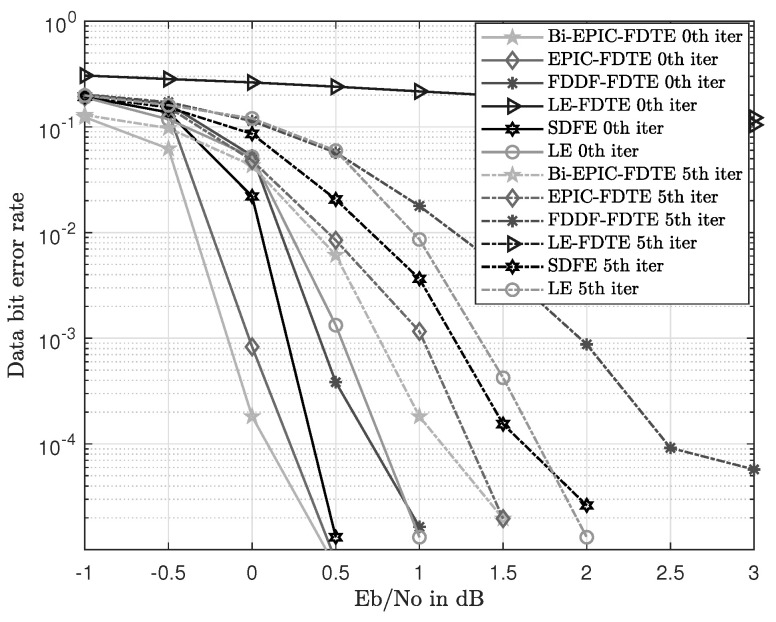
BER comparison with different equalizations in the environment of MACE’10.

The BER curves show the recovery ability of the received signals. The range of SNR is from −1 dB to 3 dB with 0.5 dB intervals. The BER curves are simulated and shown in Figure 10 and Figure 11. With the increase in SNR, the BER performance of all equalization schemes is improved significantly.

In Figure 10, the IFDTE has 1, 0.5, and 0.4 dB gains compared with FDDF-FDTE, Exact-LE, and SDFE, respectively, under the time-varying UWAC channel of SPACE’08 with a BER of $10^{-3}$. In this simulation, the proposed Bi-EPIC-FDTE obtains the best BER performance of $3.35\times 10^{-2}$, $4.74\times 10^{-4}$, and $1.98\times 10^{-5}$ at SNR of 0 dB, 0.5 dB, and 1 dB among all contrasted schemes, respectively. In Figure 11, LE-FDTE in the time-varying UWAC channel of MACE’10 cannot converge well in the set SNR under the same BER of $10^{-3}$. The IFDTE has 0.5, 0.4, and 0.2 dB gains compared with the Exact-LE, FDDF-FDTE, and SDFE, respectively. In this simulation, the proposed Bi-EPIC-FDTE obtains the best BER performance of $4.12\times 10^{-2}$, $1.87\times 10^{-4}$, and $7.98\times 10^{-6}$ at SNR of −0.5 dB, 0 dB, and 0.5 dB among all contrasted schemes, respectively. The result can be explained as follows. The IFDTE estimates the a posteriori probability iteratively with the Gaussian distribution in (13), (16), and (17) to estimate the actual a posteriori distribution iteratively with moment matching. Thus, the IFDTE outperforms its counterparts in terms of other equalization schemes.

In Figure 10 and Figure 11, the bidirectional structure of equalization provides 0.25 and 0.2 dB for the IFDTE in SPACE’08 and MACE’04 with bidirectional gain. This finding is because the bidirectional structure of the IFDTE obtains bidirectional diversity, and the performance of the Bi-IFDTE outperforms the IFDTE. Therefore, the IFDTE outperforms the conventional turbo equalization, and the bidirectional structure improves the performance of the IFDTE.

Table 4 summarizes the running time of different equalization schemes during numerical simulations. The time-domain equalization runs longer than the frequency domain one. Although the running time of the IFDTE equalization is longer than other FDTEs, it can still be accepted owing to its better performance. Thus, accompanied by the analyses in Section 3, the proposed scheme compromises the computational complexity and the equalization performance.

In fact, our research work has included a rather comprehensive comparison with typical existing equalization schemes in UWACs to highlight the novelty and superiority of the proposed approach. And the comprehensive comparison can be seen from the simulations in Figure 5, Figure 6, Figure 10 and Figure 11 with well-established methods, including the TDTE and FDTE and their extensions. In addition, the simulation of the MSE performance curve in Figure 4 and the EXIT charts in Figure 8 and Figure 9 have further verified the good performance of the proposed scheme. Therefore, there are adequate data to verify the significant performance of the proposed scheme. However, there are also potential challenges or scenarios where the proposed method may not be as effective. This mainly relates to the Cramer’s Rao criterion, which all estimation algorithms must obey. If the UWAC channel is time variance, and the channel parameters change within one block length of the channel codes employed in the turbo equalization, the estimation cannot be obtained and the proposed algorithm is not effective either.

## 5. Conclusions

In this paper, a scheme employing the IFDTE with iterative channel estimation is proposed. The FDTE has a lower complexity and slightly weaker performance in terms of signal recovery than that of the TDTE in UWACs. A channel estimation combined with the SZA-IPNLMS and SM SZA-DIPNLMS is adopted, to achieve more accurate channel estimation compared with other sparse channel estimations. The proposed IFDTE is adopted to obtain an accurate a posteriori probability of symbols iteratively with the EP. The bidirectional structure is exploited to accelerate the convergence of turbo equalization. The innovations are listed as follows: precise sparse adaptive channel estimating using the selective zero attracting penalty term; computational complexity reduction with minimal performance loss using the selective update strategy; and high-quality recovery of UWAC signals using the IFDTE and bidirectional equalization structure. The simulation results of the EXIT charts and BERs show that the proposed scheme achieves faster convergence and better performance than those of other traditional equalization schemes with acceptable complexity. The proposed IFDTE obtains 1.9 and 0.5 dB performance gains, when compared with those of the IPNLMS and $l_{0}$-IPNLMS at a BER of $10^{-3}$. It also outperforms the traditional TDTE and FDTE in UWACs by 0.5 and 1 dB, and 0.5 and 0.4 dB at the same BER of $10^{-3}$ in the environment of SPACE’08 and MACE’04, respectively. Therefore, the proposed scheme can be efficiently used in UWACs, such as in underwater sensor networks, underwater vehicles, and other similar applications.

## Figures and Tables

**Table 1:** Procedures of the IFDTE with iterative channel estimation.

Procedures of the IFDTE with Iterative Channel Estimation
(Step 1). If no soft feedback exists, the SZA-IPNLMS is utilized to estimate the CSI in terms of the training sequence in (2), otherwise, the SZA-DIPNLMS is utilized to update the CSI in terms of the soft feedback in (37). (Step 2). Calculate the a posteriori probability by using (15), and the a posteriori probability is continuously optimized by using (16)–(25) based on the EP. Repeat Step 2 until the maximum self-iteration for the EP is reached. (Step 3). On the basis of the estimated a posteriori information, the extrinsic information of equalization is calculated by using (24) and is then inputted into the decoder. (Step 4). The decoder generates the extrinsic information in terms of the log-MAP criterion in (27). If the maximum turbo equalization is not reached, the outputted extrinsic information of the decoder is regarded as the a priori information for equalization. Return to Step 1, otherwise, the decoder outputs the transmitted bits.

**Table 2:** Complexity of equalizing symbols for different turbo equalizers.

Algorithm	Equalizer Vector	Symbol Estimation	A Posteriori Moments	Conditional Moments
FDDF-FDTE [11]	8MK	2MK+ $K\log_{2}K/2$	-	$\frac{2^{Q}+1}{2}$
Proposed IFDTE	5MKS	(2MK+ $K\log_{2}K/2)S$	$2^{Q+1}S$	-
SDFE [6]	$\left(M^{3}N_{s}^{3}/2+MN_{s}+\right.$ $\left(M+M^{2}\right)(N_{s}+$ ${L-2)}^{2})K$	$(N_{s}+N_{2}+L)MK$	-	$\frac{2^{Q}+1}{2}$
LE [20]	$\left(M^{3}N_{s}^{3}/2+\right.$ $\left(M+M^{2}\right)(N_{s}$ ${+L)}^{2})K$	$M(N_{s}+L)K$	-	$\frac{2^{Q}+1}{2}$

**Table 3:** Parameters of SPACE’08 and MACE’10 in UWAC simulations.

Experiment	SPACE’08	MACE’10
Carrier Frequency (kHz)	13	13
Bandwidth [kHz]	9	5
The Depth of Water [m]	10	100
Transmitter Height [m]	4	80
Receiver Height [m]	2, 6	80, 70, 60, 50
Distance between Tx and Rx [km]	1	0.5–4
Relative Velocity between Tx and Rx [m/s]	0	1
Spread Factor	1.7	
Bottom Density [g/m^3^]	1.269	
Auto Regression (AR) Factor	0.9	
Sampled Delay Points	148	

**Table 4:** Statistics on CPU running time for different equalizers.

Algorithm	FDE-FDTE	FDDF-FDTE	EPIC-FDTE	SDFE	LE
CPU times (s)	0.0083	0.0114	0.0541	0.2963	0.2455

## Data Availability

Not applicable.

## Abbreviations

BER	Bit error rate
Bi-IFDTE	Bidirectional IFDTE
CSI	Channel status information
CIR	Channel impulse response
CE	Channel estimation
CS	Compress sensing
DIPNLMS	Differential IPNLMS
EP	Expectation propagation
EXIT	Extrinsic information
FDTE	Frequency domian turbo equalization
FDDF	Frequency domain decision feedback
ICE	Iterative channel estimation
IFDTE	Improved FDTE
ISI	Inter-symbol interference
IPNLMS	Improved proportionate NLMS
LE	Linear equalization
MAP	Maximum a posteriori probability
MMSE	Minimum mean square error
MSE	Mean square error
NLMS	Normal least mean square
Prob.	Probability
SM	Set-membership
SZA	Selective zero-attracting
SIMO	Single-input multiple-output
TDTE	Time-domain turbo equalization
UWACs	Underwater acoustic communications
